# *In vitro* blood cell responsiveness to IFN-α predicts clinical response independently of IL28B in hepatitis C virus genotype 1 infected patients

**DOI:** 10.1186/1479-5876-12-206

**Published:** 2014-07-21

**Authors:** Nollaig M Bourke, Mary-Teresa O’Neill, Shahzad Sarwar, Suzanne Norris, Stephen Stewart, John E Hegarty, Nigel J Stevenson, Cliona O’Farrelly

**Affiliations:** 1 School of Biochemistry and Immunology, Trinity Biomedical Sciences Institute, Trinity College Dublin, Dublin 2, Ireland; 2 Liver Centre, Mater Misericordiae University Hospital, Dublin 7, Ireland; 3 Liver Unit, St. Vincent’s University Hospital, Dublin 4, Ireland; 4 Hepatology Unit, St. James’s Hospital, Dublin 8, Ireland; 5 School of Medicine, Trinity College, Dublin 2, Ireland

**Keywords:** Interferon stimulated genes, Protease inhibitors, Host predictive markers, IL28B genotype

## Abstract

**Background:**

Treatment with interferon-alpha (IFN-α) and ribavirin successfully clears hepatitis C virus (HCV) infection in 50% of patients infected with genotype 1. Addition of NS3-4A protease inhibitors (PIs) increases response rates but results in additional side effects and significant economic costs. Here, we hypothesised that *in vitro* responsiveness of peripheral blood mononuclear cells (PBMCs) to IFN-α stimulation would identify patients who achieved sustained virological response (SVR) on dual therapy alone and thus not require addition of PIs.

**Methods:**

PBMCs were isolated from HCV infected patients (n = 42), infected with either HCV genotype 1 or genotype 3, before commencing therapy and stimulated *in vitro* with IFN-α. Expression of the IFN stimulated genes (ISGs) PKR, OAS and MxA was measured and correlated with subsequent treatment response and IL28B genotype.

**Results:**

Genotype 1 infected patients who achieved SVR had significantly higher pre-treatment expression of PKR (p = 0.0148), OAS (p = 0.0019) and MxA (p = 0.0019) in IFN-α stimulated PBMCs, compared to genotype 1 infected patients who did not achieve SVR or patients infected with genotype 3, whose *in vitro* ISG expression did not correlate with clinical responsiveness. IL28B genotype (rs12979860) did not correlate with endogenous or IFN-α stimulated ISG responsiveness.

**Conclusions:**

*In vitro* responsiveness of PBMCs to IFN-α from genotype 1 infected patients predicts clinical responsiveness to dual therapy, independently of IL28B genotype. These results indicate that this sub-group of HCV infected patients could be identified pre-treatment and successfully treated without PIs, thus reducing adverse side effects and emergence of PI resistant virus while making significant economic savings.

## Background

The standard of care for patients infected with hepatitis C virus (HCV) for the past decade has consisted of dual therapy with the anti-viral cytokine, interferon-alpha (IFN-α) and the nucleoside analogue, ribavirin. IFN-α induces anti-viral immunity by upregulating hundreds of IFN stimulated genes (ISGs) [1], including many with potent direct and indirect anti-viral activity [2]. Response rates to therapy are highly variable, with patients infected with genotype 1 (G1) having sustained virological response (SVR) rates of less than 50%, whereas genotype 3 (G3) infected patients can achieve SVRs of up to 82% [3].

Increased insight into the viral life cycle of HCV has led to development of several new directly acting anti-viral agents (DAAs), including NS3-4A protease inhibitors (PIs). Several PIs, including telaprevir and boceprevir, are administered in combination with standard IFN-α/ribavirin treatment. Triple therapy increases response rates from less than 50% to 75% in some G1 infected cohorts [4,5]. However, PIs are expensive and associated with significant additional side effects, such as anaemia and rash, and emergence of drug resistant variants, a major challenge in cases of non-compliance with therapy. Identification of patients with a high probability of obtaining an SVR to dual therapy would obviate the need for additional PIs and alleviate these issues.

Multiple efforts have been made to accurately predict response to therapy using viral and host characteristics. Viral predictive markers include viral load and genotype, while host predictive markers include age, sex, race, and liver fibrosis stage [6]. Elevated serum levels of the chemokine CXCL10 have also been reported to be associated with non-response to IFN-α therapy [7]. Additionally, a single nucleotide polymorphism (SNP), rs12979860, in the recently described IFNλ4 gene [8] is highly predictive of response [9,10]. The major C allele correlates strongly with viral clearance particularly in patients infected with HCV G1. Nevertheless, no single marker or combination of markers accurately predicts patient response in individual cases.

We hypothesised that *in vitro* responsiveness to IFN-α would predict clinical responsiveness to dual therapy. Hepatic ISG expression is elevated pre-treatment in patients who fail to achieve SVR [11] and has been shown to be a stronger predictor of response than IL28B genotype [12]. However, liver biopsy is an invasive procedure with associated risks and has limited value as a prognostic tool. Leukocytes are sensitive responders to IFN-α and provide a more accessible alternative, requiring just a peripheral blood sample. In fact, *in vivo* upregulation of ISGs in PBMCs following therapeutic IFN-α is similar to ISG upregulation *in vitro* following IFN-α stimulation [13], suggesting *in vitro* PBMC responsiveness may indeed be an accurate reflection of *in vivo* clinical response.

IFN-α activates the JAK-STAT signalling pathway, leading to upregulation of over 500 ISGs [14]. PKR, OAS and MxA are three well-characterised ISGs, strongly induced by IFN-α in PBMCs, which have direct anti-viral action. Activation of PKR by virus results in inhibition of protein translation, including inhibition of viral mRNA translation through phosphorylation of the alpha subunit of eukaryotic protein synthesis initiation factor 2 (eIF2α) [15]. Indeed, HCV has evolved several mechanisms to block the action of this important regulator of translation [16,17]. OAS is an IFN regulated activator of latent ribonuclease RNase L, which is triggered by activated OAS to directly cleave RNA, including HCV RNA, thus destroying viral RNA products and producing pathogen associated molecular patterns (PAMPs) that further stimulate innate immune activity [18,19]. MxA recognises viral nucleocapsids and renders them redundant by wrapping around the viral structure and forming MxA/nucleocapsid oligomers [20]. MxA may also direct nucleocapsids to alternative sites in the cytoplasm, where they are not functional for RNA synthesis and likely to be immobilised and subsequently degraded [2]. Interestingly, we have demonstrated that the core protein of HCV co-localises with MxA in a granular pattern in the cytoplasm of cells, a phenomenon that is potentiated with the co-treatment of IFN-α and ribavirin [21]. Hepatic expression of MxA is a known predictor of response to IFN-α therapy [22,23]. Because of their role as key mediators of IFN-α-induced antiviral activity, PKR, OAS and MxA were chosen as potential indicators of IFN-α treatment responsiveness in this prospective study.

Prior to treatment, we measured *in vitro* responsiveness of PBMCs to IFN-α stimulation by quantifying PKR, OAS and MxA expression levels in IFN-α treated PBMCs isolated from HCV infected patients. We correlated ISG expression with the patient’s subsequent clinical response to therapy and with other factors including IL28B genotype.

## Methods

### Study population

Patients (n = 41) from St. Vincent’s University Hospital (SVUH) and St. James’s Hospital Dublin (SJH), who were mono-infected with HCV, were recruited. Written consent was obtained from each patient and ethical approval was obtained from the ethics and medical research committee at SVUH and the research ethics committee at SJH, in accordance with the ethical guidelines of the 1975 Declaration of Helsinki. Patients were treated with pegylated IFN-α2a or IFN-α2b in combination with ribavirin as previously described [24]. Patients who were HCV-RNA negative at week 4 of treatment were termed rapid virological responders (RVR); patients with a 2 log drop in viral RNA by week 12 were termed early virological responders (EVR); HCV-RNA –ve patients at the end of treatment were classed as having an end-of-treatment response (EOT); patients who were HCV-RNA 6 months post treatment achieved SVR; and those who were HCV-RNA at EOT but had viral breakthrough 6 months post treatment were termed relapsers.

### PBMC preparation and stimulation

Blood samples were collected in lithium heparin tubes and PBMCs were freshly isolated by density centrifugation using Ficoll-Paque separation medium (GE Healthcare, Sweden). 2 × 10^6^ PBMCs per ml of RPMI, supplemented with 10% FCS and 250 U/ml penicillin, 250 μg/ml streptomycin, were cultured at 37°C, and stimulated fresh with 100 IU or 1000 IU pegylated IFN α-2a (Roche, Switzerland) for 2 and 4 hours. PBMCs were immediately lysed in Trizol reagent (Invitrogen, USA) following stimulation, thus preserving the RNA and stored at -80°C until analysis.

### RNA Extraction and Quantitative Real-time PCR (qRT-PCR)

RNA was extracted according to manufacturer’s instructions and reverse transcribed using Omniscript (Invitrogen). qRT-PCR using SyBr green was performed on an MX3000P® system (Stratagene Corp, USA) using the following cycling parameters: 95°C for 30 sec, 60°C for 1 min and 72°C for 30 sec. Following geNORM analysis of a panel of housekeepers, the most stable housekeeping gene was found to be ribosomal protein 15 (RPS15) [25]. Gene amplifications were normalised to RPS15 and expressed on a log scale [26]. Primers are shown in Table 1.

**Table 1:** Intron spanning primers for qRT-PCR analysis

**Gene**	**Sense**	**Antisense**
**RPS15**	CGGACCAAAGCGATCTCTTC	CGCACTGTACAGCTGCATCA
**MxA**	GGTGGTGGTCCCCAGTAATG	ACCACGTCCACAACCTTGTCT
**OAS**	GAAGCCCTACGAAGAATGTCAGA	TCGGAGTTGCCTCTTAAGACTGT
**PKR**	TCTCAGCAGATACATCAGAGATAAATTCT	AGTATACTTTGTTTCTTTCATGTCAGGAA

### IL28B SNP analysis

Rs12979860 genotype was determined from patient blood or serum samples using the LightMix Kit IL28B (Roche/Tib MolBiol).

### Statistical analysis

Paired samples were analysed using Wilcoxon matched-pairs rank tests; for unpaired samples, non-parametric Mann–Whitney U tests and Fisher’s exact test were used. Area under the curve (AUC) analysis was calculated from receiver operating characteristics (ROC) curve. P values <0.05 were considered statistically significant.

## Results

### Patient characteristics

Forty-one patients with chronic HCV infection were studied prospectively, the majority of whom had been infected via IV drug use (Table 2). Eighteen patietns were infected with genotype1 (G1) and 23 with genotype 3 (G3). Numbers of patients, gender ratios and ages were similar in both groups. Demographic, clinical, virological and genetic features were also similar. Liver enzyme levels (ALT p = 0.34, AST p = 0.5), viral load (p = 0.31) and IL28B genotype (CC p = 0.5, CT/TT p = 0.36) were similar in both groups. Circulating lymphocytes tended to be lower in G1 infected patients (1.8 × 10^3^/μl) when compared with G3 infected patients (2.3 × 10^3^/μl) but this difference was not significant (p = 0.06). Total white cell counts, neutrophil and monocyte counts were similar in both patient cohorts. Of the 41 patients recruited, 36 successfully completed therapy (G1 n = 16, G3 n = 20). Surprisingly, in this study there was no significant difference in response rates between G1 or G3 infected patients recruited (Table 3; RVR 42% vs 57% respectively, p = 0.54; EVR 83% vs 90% p = 0.64; EOT 72% vs 86% p = 0.68; SVR 56% vs 65% p = 0.74).

**Table 2:** Clinical features

**Feature**	**Genotype 1**	**Genotype 3**	P value*
Patients (n)	18	23	0.7^§^
Males, n (%)	13 (68)	15 (65)	1^§^
Females, n (%)	6 (32)	8 (35)	
Age (years -/+SD)	44.6 -/+13.2	43.2 -/+10.2	0.19^¶^
Risk factors, n (%)			
Injecting drug use	7 (39)	12 (52)	0.53^§^
Blood transfusion	4 (22)	2 (9)	0.03^§^
Anti-D immunoglobulin	4 (22)	0 (0)	0.38^§^
Not determined	3 (17)	9 (39)	0.17^§^
Liver enzyme levels			
ALT (IU/L -/+SD)	112.8 -/+122.6	107.4 -/+86.3	0.34^¶^
AST (IU/L -/+SD)	66.6 -/+54.8	69.2 -/+59.5	0.5^¶^
Leukocyte counts			
WCC (x 10^9^/L -/+SD)	5.7 -/+2	6.4 -/+2.5	0.3^¶^
Neutrophils (x10^3^/μl)	3.3 -/+1.4	3.4 -/+1.7	0.99^¶^
Lymphocytes (x10^3^/μl)	1.8 -/+0.6	2.3 -/+1	0.06^¶^
Monocytes (x10^3^/μl)	0.46 -/+0.13	0.6 -/+0.3	0.17^¶^
Viral load (x10^6^ IU/ml -/+) SD)	5.6 -/+10.8	7.5 -/+1.3	0.31^¶^
IL-28B genotype n (%)			
CC	4 (22)	8 (35)	0.5^§^
CT/TT	10 (56)	9 (39)	0.36^§^
Not determined	4 (22)	6 (26)	1^§^

*Comparison between G1 and G3 infected patients.

^§^Fisher’s exact test.

^¶^Mann–Whitney *U* test.

*ALT* alanine aminotrasferase, *AST* aspartate aminotransferase, *WCC* white cell count.

**Table 3:** Clinical response to treatment

**Therapeutic outcome**	**Genotype 1**	**Genotype 3**	**P value**^**§**^
RVR (%)	8 (42)	13 (57)	0.54
EVR (%)	15 (83)	20 (90)	0.64
EOT (%)	13 (72)	19 (86)	0.68
SVR (%)	10 (62.5)	13 (65)	0.74
Relapse (%)	3 (23)	6 (33)	1

^§^Fisher’s exact test.

### Pre-treatment ISG expression in PBMCs predicts SVR in G1, but not G3, infected patients

**Figure 1:**
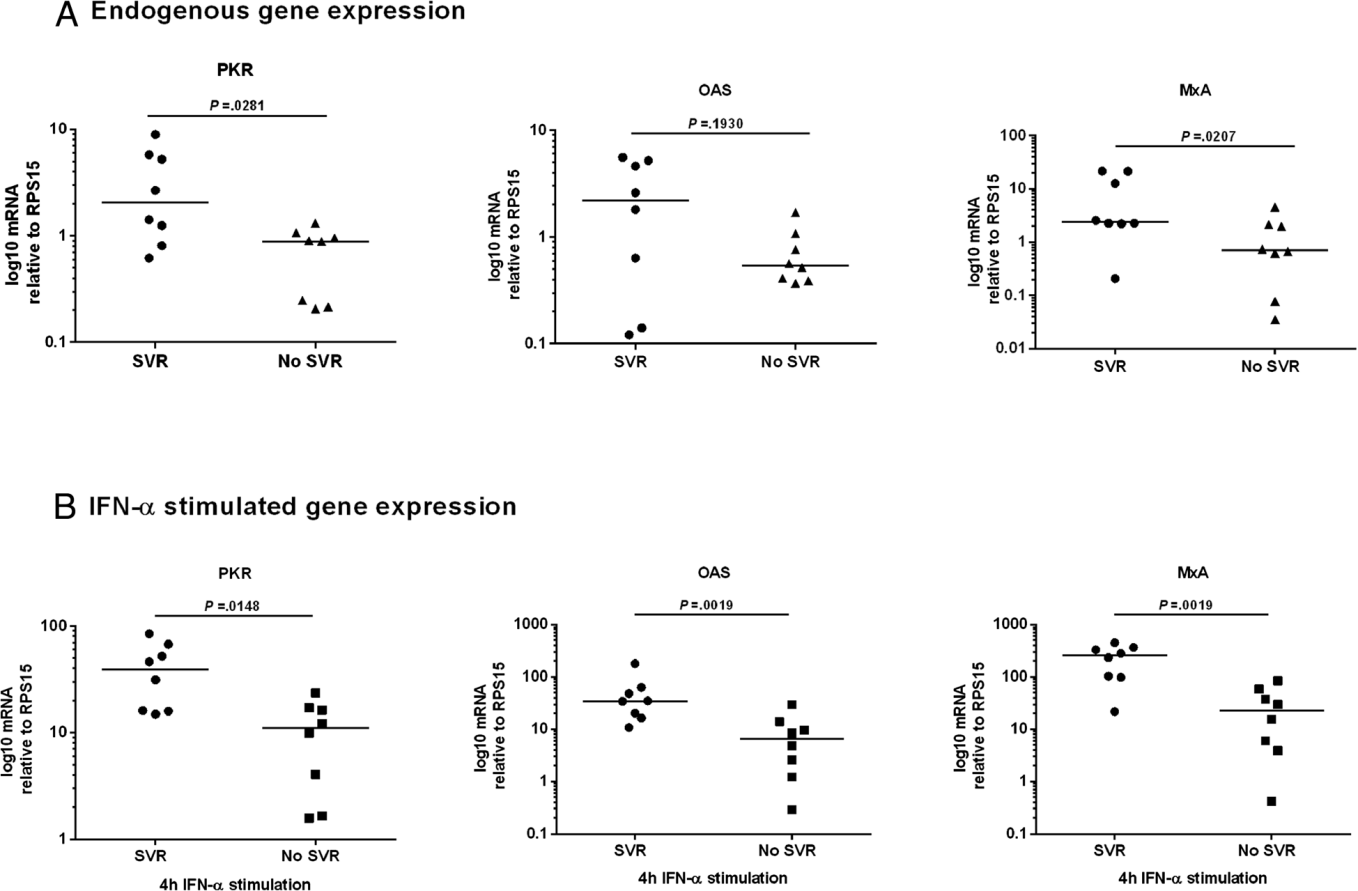
**G1 infected patients who achieve SVR have higher ISG expression pre-treatment than those without SVR.** PBMCs were isolated from G1 infected patients pre-treatment and stimulated with IFN-α. qRT-PCR was used to quantify **(A)** endogenous and **(B)** IFN-α stimulated induction of ISGs. Gene expression was normalised to expression of RPS15 and expressed on a log scale. Data were analysed using Mann–Whitney U-tests.

**Figure 2:**
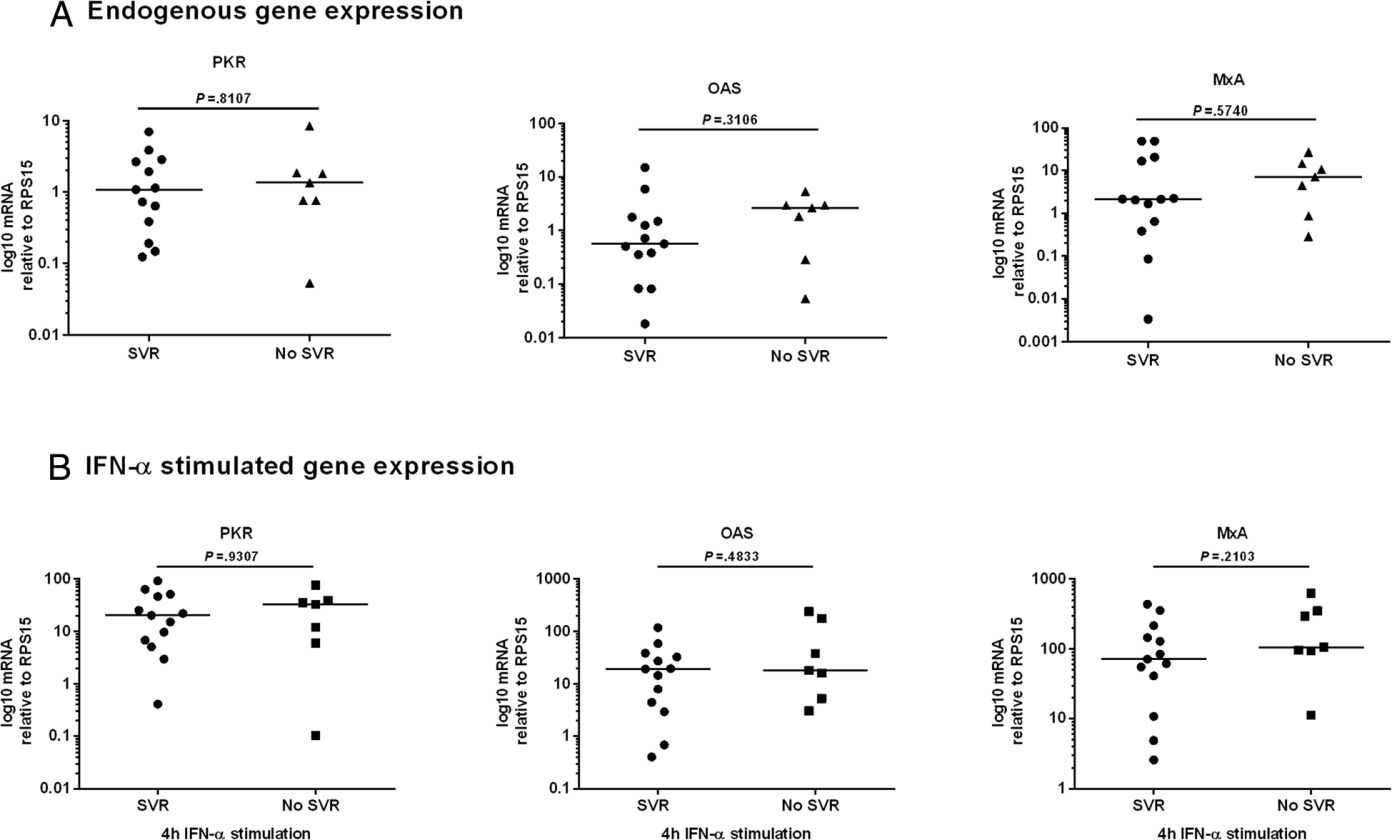
**ISG expression is not predictive of treatment response in patients infected with HCV g3.** PBMCs were isolated from G3 infected patients pre-treatment and stimulated with IFN-α. qRT-PCR was used to quantify **(A)** endogenous and **(B)** IFN-α stimulated induction of ISGs. Gene expression was normalised to expression of RPS15 and expressed on a log scale. Data were analysed using Mann–Whitney U-tests.

To determine whether *in vitro* responsiveness of PBMCs to IFN-α could predict clinical response to therapeutic IFN-α, PBMCs were isolated from patients before they commenced therapy, stimulated *in vitro* with IFN-α and ISG expression was quantified. G1 infected patients who successfully achieved SVR following treatment had significantly higher induction of PKR (p = 0.0148), OAS (p = 0.0019) and MxA (p = 0.0019; Figure 1B) following stimulation with IFN-α compared to G1 infected patients who failed to achieve SVR. As well as having higher induction of these ISGs following *in vitro* IFN-α stimulation, G1 infected patients who achieved SVR also had increased endogenous levels of PKR and MxA (p = 0.0281 and p = 0.0207 respectively; Figure 1A). Conversely, pre-treatment endogenous or IFN-α induced ISG expression was not consistently higher in G1 infected patients who achieved RVR (Additional file 1: Figure S1), EVR (Additional file 1: Figure S2) or EOT response (Additional file 1: Figure S3) compared to those who did not. Therefore, in G1 infected patients, enhanced ISG expression only correlated with SVR and not with other on-treatment response rates. In contrast to G1 infected patients, G3 infected patients showed no correlation with either endogenous ISG expression (PKR p = 0.8107, OAS p = 0.3106, MxA p = 0.5740; Figure 2A) or IFN-α stimulated ISG expression (PKR p = 0.9307, OAS p = 0.4833, MxA p = 0.2103; Figure 2B) and achieving an SVR.

To determine the predictive value of IFN-α responsiveness in G1 infected patients, ROC curves were calculated based on ISG expression following stimulation with IFN-α. PKR expression had an AUC of 0.86 (p = 0.016) while both OAS and MxA each had an AUC of 0.94 (p = 0.003) (Additional file 1: Table S1). We used predictive values of gene cut-off expression levels (generated from the AUC analysis) to evaluate which patients would respond to dual therapy and thus not require additional PIs. Of all three genes examined, MxA had the strongest predictive power, with a PPV of 100%, a NPV of 88.9% and a likelihood ratio of 8 based on a cut-off of 91.2, despite the low numbers of patients in the analysis.

Univariate analysis was used to determine whether other pre-treatment characteristics were predictive of treatment response in our G1 and G3 infected cohorts (Table 4). In G1 infected patients, PKR, OAS and MxA induction was the only significant predictor of SVR. Lack of response to therapy was not associated with altered numbers of white blood cells. In G3 infected patients, achieving SVR was significantly associated with lower pre-treatment viral load (p = 0.045) and younger age (p = 0.03). Achieving RVR (p = 0.06), and high pre-treatment lymphocyte numbers (p = 0.07) were also close to being significantly associated with SVR in this cohort.

**Table 4:** Univariate analysis of predictors of SVR in g1 and g3 infected patients

**Genotype 1 infected patients**	**SVR (n = 8)**	**No SVR (n = 8)**	**P value**
Age (years -/+SD)	40.9 -/+4.2	47.3 -/+5.6	0.4^¶^
Sex (m/f)	6/2	4/4	0.61^§^
IL28B genotype (CC/CT or TT)*	5/3	5/1	0.58^§^
Fibrosis	2.5 -/+0.9	1.7 -/+0.6	0.59^¶^
RVR (Y/N)	4/4	2/6	0.61^§^
Viral load (x10^6^)	2.1 -/+1.1	5.6 -/+3.1	0.65^¶^
Liver enzyme levels			
ALT	87 -/+26.6	44.1 -/+8.6	0.57^¶^
AST	162.3 -/+58.2	53.3 -/+8.9	0.19^¶^
Leukocyte counts			
White cell count	5.6 -/+1	5.9 -/+0.5	0.56^¶^
Neutrophils	3.2 -/+0.7	3.3 -/+0.4	0.8^¶^
Lymphocytes	1.8 -/+0.3	1.9 -/+0.2	0.68^¶^
Monocytes	0.5 -/+0.1	0.4 -/+0.05	0.72^¶^
ISG expression			
PKR endogenous	3.3 -/+1.1	0.7 -/+0.1	**0.028**^**¶**^
OAS endogenous	2.6 -/+0.8	0.7 -/+0.2	0.19^¶^
MxA endogenous	8.1 -/+3.2	1.3 -/+0.5	**0.021**^**¶**^
PKR induction	41.1 -/+9.2	10.8 -/+2.8	**0.015**^**¶**^
OAS induction	51 -/+19.3	8.8 -/+3.4	**0.002**^**¶**^
MxA induction	235 -/+52.5	29.7 -/+10.6	**0.002**^**¶**^
**Genotype 3 infected patients**	**SVR (n = 13)**	**No SVR (n = 7)**	**P value**
Age (years -/+SD)	39.6 -/+1.9	47 -/+2.4	**0.03**^**¶**^
Sex (m/f)	7/6	5/2	0.64^§^
IL28B genotype (CC/CT or TT)*	4/5	4/3	1^§^
RVR (Y/N)	10/3	2/5	**0.06**^**§**^
Viral load (x10^6^)	7.8 -/+3.8	9.6 -/+3.6	**0.045**^**¶**^
Liver enzyme levels			
ALT	93 -/+17	147 -/+38	0.24^¶^
AST	58.2 -/+9.8	109 -/+25.6	0.13^¶^
Leukocyte counts			
White cell count	6.6 -/+0.7	5.8 -/+0.8	0.37^¶^
Neutrophils	3 -/+0.5	3.1 -/+0.5	0.76^¶^
Lymphocytes	2.7 -/+0.3	1.9 -/+0.2	0.07^¶^
Monocytes	0.6 -/+0.1	0.5 -/+0.1	0.71^¶^
ISG expression			
PKR endogenous	2.1 -/+0.5	2 -/+1	0.81^¶^
OAS endogenous	2.2 -/+1	2 -/+0.6	0.31^¶^
MxA endogenous	11.4 -/+4.4	8.1 -/+3.2	0.57^¶^
PKR induction	29.1 -/+6.9	29.5 -/+9.6	0.93^¶^
OAS induction	23.9 -/+8	70.6 -/+36.8	0.48^¶^
MxA induction	126 -/+34	226.4 -/+81.2	0.21^¶^

*RVR* rapid virological response, *ALT* alanine aminotrasferase, *AST* aspartate aminotransferase.

^*^Data not available for all patients included in study.

^§^Fisher’s exact test.

^¶^Mann–Whitney *U* test.

### Rs12979860 genotype does not affect ISG expression in PBMCs from HCV infected patients

**Figure 3:**
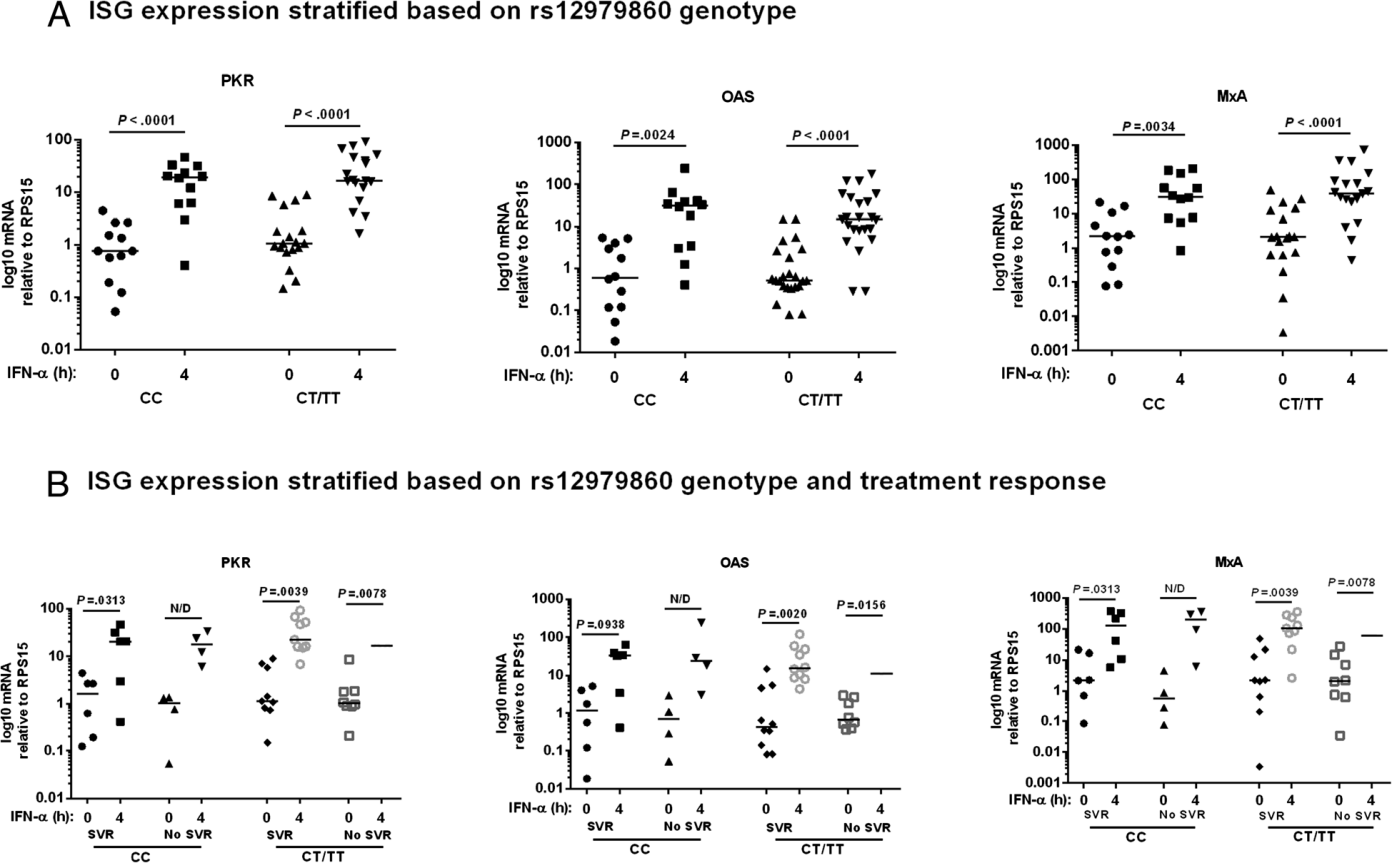
**rs12979860 genotype is independent of ISG expression in PBMCs from HCV infected patients.** PBMCs were isolated from HCV infected patients pre-treatment and stimulated with IFN-α. qRT-PCR was used to quantify endogenous and IFN-α induced expression of ISGs. ISG expression was stratified based on **(A)** rs12979860 genotype and **(B)** rs12979860 genotype and treatment response. Gene expression was normalised to expression of RPS15 and expressed on a log scale. Each dot represents one sample and median gene expression is shown. Data were analysed using Wilcoxon matched-pairs rank test and Mann–Whitney U-tests; N/D = not determined due to low n number.

**Figure 4:**
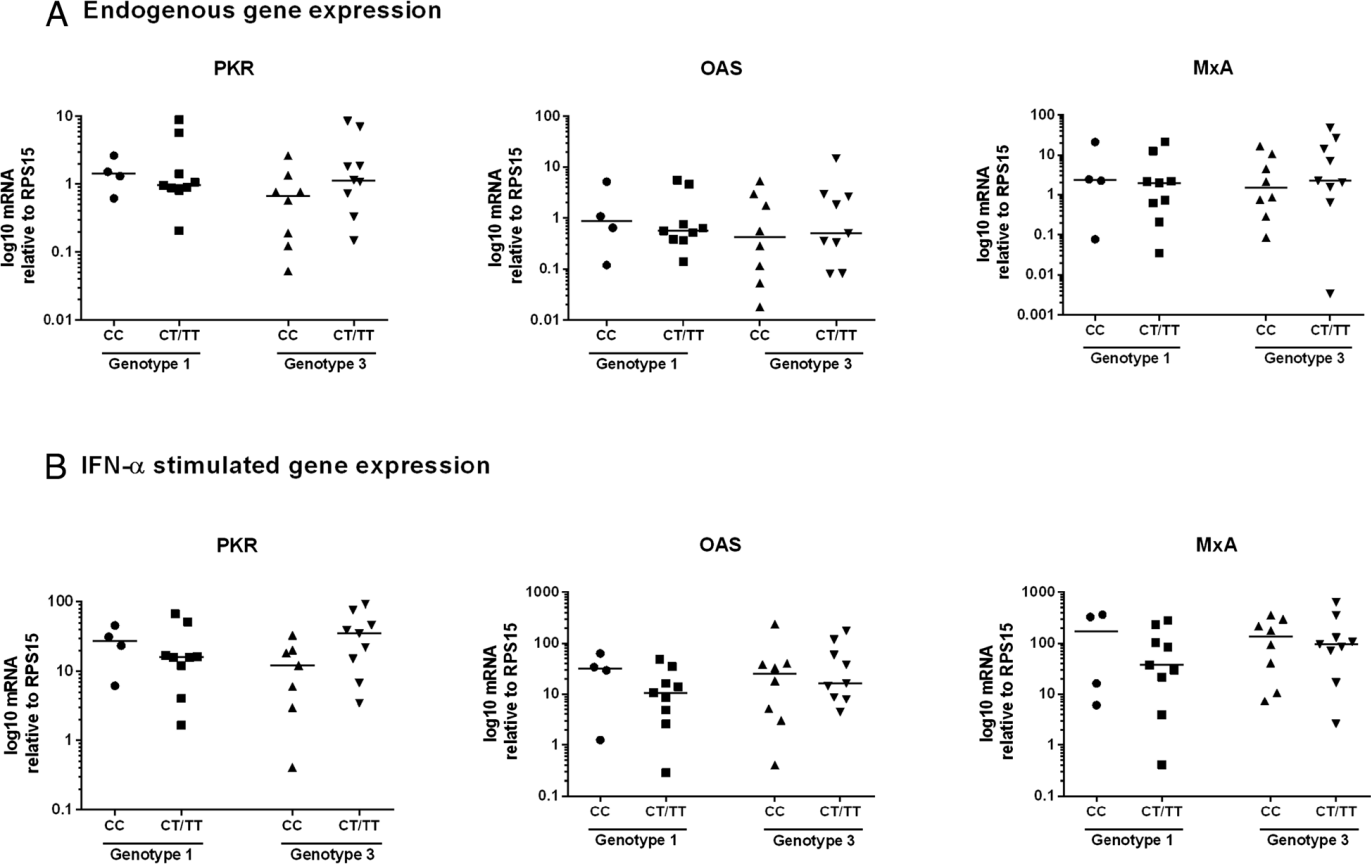
**ISG expression, stratified based on IL28B genotype, is similar between G1 and G3 infected patients.** PBMCs were isolated from HCV infected patients prior to them commencing IFN-α therapy and were stimulated in vitro with 1000 IU of IFN-α for 4 h. qRT-PCR was used to quantify endogenous and IFN-α stimulated expression of ISGs. ISG expression was stratified based on rs12979860 genotype and **(A)** endogenous and **(B)** IFN-α stimulated ISG expression was measured. All gene expression was normalised to expression of the internal control gene RPS15 and expressed on a log scale. Each dot represents one sample and median gene expression is shown. Data were analysed using Wilcoxon matched-pairs rank test and Mann–Whitney U-tests.

In other studies, the SNP (rs12979860) in the IFNλ4 gene, some distance from the IL28 genes, is highly predictive of treatment response [10,27]. ISG expression in liver tissue from HCV infected patients has been shown to correlate with rs12979860 genotype [12]. We therefore investigated whether rs12979860 genotype influenced ISG expression in PBMCs from HCV infected individuals in our study. IFN-α stimulation of PBMCs led to robust upregulation of ISG expression, regardless of whether patients carried the minor T allele for rs12979860 (Figure 3A), demonstrating that *in vitro* induction of IFN-α stimulated ISGs in PBMCs was independent of IL28B genotype. Additionally, rs12979860 genotype did not influence endogenous ISG levels in PBMCs. When ISG expression in patients who achieved SVR was compared to those who did not, and stratified based on rs12979860 genotype, there were no significant differences observed between patients with CC or CT/TT genotype (Figure 3B). There was also no difference in ISG expression and IL28B genotype between G1 and G3 infected patients (Figure 4).

## Discussion

Approximately 50% of HCV infected patients infected with G1 achieve an SVR with IFN-α and ribavirin therapy and do not actually need additional PIs in their treatment regime. However, it has it has not hitherto been possible to identify responsive patients prior to commencing treatment. Here, we hypothesised that *in vitro* analysis of immune cell responsiveness to IFN-α stimulation prior to treatment would predict clinically responsive patients. In this prospective study, we found that G1 infected patients who achieved SVR could indeed be identified prior to treatment by the significantly greater ISG induction in their PBMCs following *in vitro* IFN-α stimulation than in PBMCs from G1 infected patients who did not achieve SVR. Interestingly, this finding seems to be exclusive to patients infected with GI as the clinical response of G3 infected patients could not be predicted pre-treatment. IL28B genotype did not influence ISG expression in PBMCs nor *in vitro* IFN-α stimulated responses and there was no difference in ISG expression between patients who achieved SVR or did not achieve SVR among the different IL28B genotypes.

The development of DAAs is an exciting advance in treating HCV infection and offers many genotype 1 infected patients who would not respond to standard IFN-α therapy, additional options for treatment. However, currently licensed PIs are given in combination with dual IFN-α therapy, thus significantly increasing costs and introducing additional significant side effects. Trials involving DAAs in “IFN free” regimes are extremely promising for certain groups of patients, however IFN-α therapy still boasts a lack of drug-drug interactions, relatively low cost, lack of effect of viral resistant mutants and a long clinical track record [28]. Crucially, the cost of DAAs will prevent their large scale distribution in low and middle income countries, which have the highest rates of HCV infection, for the foreseeable future. Therefore, identification of subgroups of patients who would not require additional PIs would make significant economic savings.

Several predictors of response to dual therapy have previously been described including demographic, genetic and intrahepatic markers [3]. Studies have shown hepatic ISG expression to be a strong predictor of treatment response [11,29]. Using microarrays, others have shown that global transcriptional responses in PBMCs following *in vivo* and *ex vivo* IFN-α stimulation are different between responders and non-responders following treatment [30,31]. We found that G1 infected patients who achieved an SVR had significantly higher ISG expression in PBMCs prior to treatment. Pre-treatment, endogenous expression of PKR and MxA was lower in PBMCs from G1 infected patients who failed to achieve an SVR, contrasting with previous reports from the liver showing elevated pre-treatment ISG expression predictive of therapeutic non-responsive [11]. A range of IFN-sensitive mechanisms may already be activated in HCV-infected liver, which harbours mixed populations of cells including epithelial and endothelial cells as well as hepatocytes and immune cells. In comparison, PBMCs provide a more accessible homogenous population for use as a resource for identifying patients who are responsive to IFN-α.

Interestingly, pre-treatment ISG expression in PBMCs from G3 infected patients could not predict SVR, yet ISG expression was strongly predictive of therapeutic response in G1 infected patients, suggesting that HCV G1 may differ significantly from G3 in its ability to target IFN-α signalling. Several HCV proteins, including the NS3-4A protease, are known inhibitors of the IFN response [32,33]. We recently found that HCV G1 degrades signal transducers and activator of transcription 1 (STAT1) and STAT3, key transcription factors activated by IFN-α signalling, and showed that this suppression was consistent across all PBMC subsets [34]. However, it is possible that HCV genotypes differentially target IFN-α signalling perhaps explaining the differences in ISG expression we observed in G1 and G3 infected patients.

Patients who have rapid decreases in viral load and achieve an RVR have a 90% chance of achieving overall SVR [35]. However, we found that pre-treatment ISG expression was not strongly associated with RVR, suggesting that ISG expression in PBMCs is independent of RVR, in accordance with a study by Sarasin-Filipowicz et al. [29]. That study did not examine SVR. Unusually, RVR was not predictive of overall SVR in our G1 infected cohort. Although ISG expression tended to be higher in patients who achieved EVR or were PCR –ve at the end of treatment compared to RVR in G1 infected patients, the strongest association seen was that between pre-treatment ISG expression and SVR.

In this study, we analysed expression of three well-defined anti-viral genes (PKR, OAS and MxA) that have been shown using microarray analysis to be upregulated in PBMCs and specific immune cell subsets, including macrophages, DCs and NK cells, following *in vitro* IFN-α stimulation [2,36-39]. IFN-α is a strong regulator of gene expression in PBMCs and Zimmerer et al. demonstrated that *in vitro* stimulation of PBMCs mimics *in vivo* effects [40]. This study also demonstrated that T cells, monocytes and NK cells all respond to *in vitro* IFN-α stimulation. Recently, a retrospective analysis of G1 infected HCV patients of mixed race demonstrated that SVR and monocyte activation in response to 24 h *in vitro* stimulation with IFN-α were negatively correlated but failed to show any correlation between IFN-α induced myeloid and plasmacytoid dendritic cell activation and therapeutic response. In our study, we found enhanced responses to 4 h *in vitro* stimulation in unseparated PBMCs from G1 infected patients was associated with SVR, indicating that duration of IFN-α stimulation is critical.

The rs12979860 SNP, located upstream of the IL28B gene and present in the novel IFNλ4 gene [8], is a robust predictor of spontaneous viral clearance and response to treatment in some cohorts [9,10]. Of note, hepatic ISG expression has also been shown to be a stronger predictor of response than IL28B genotype [12,22]. The predictive value of IL28B is increased when combined with the predictive power of other known indicators of response, such as serum CXCL10 levels [41]. Amongst our G1 infected patients, ISG expression in PBMCs was more predictive of therapeutic response than IL28B genotype which was a surprising result given the strong predictive power of this SNP in other studies [10,27]. However, it is possible that the IL28B genotype is not a strong predictor in all cohorts; indeed MxA staining in hepatic macrophages has been found to be a better predictor of SVR than IL28B genotype in a Canadian cohort [22]. Endogenous ISG expression has been shown to be higher in livers from patients carrying the minor T allele for rs12979860 [12], yet we found that in PBMCs, ISG expression was similar between patients of any genotype. Microarray studies have shown that ISG expression in PBMCs from G1 infected patients was independent of IL28B genotype [42]. In a HCV/HIV co-infected cohort of patients, induction of ISGs following 12 hours of *in vivo* stimulation with IFN-α, was independent of IL28B genotype, echoing our findings in PBMCs from HCV infected patients.

## Conclusions

*In vitro* responsiveness of PBMCs from G1 infected patients to IFN-α stimulation pre-treatment identifies patients who will achieve SVR on dual therapy. These patients do not need additional PIs to clear the virus. Reliable identification of patients who do not require triple therapy would provide significant cost benefits, reduce side effects and halt the emergence of PI resistant strains of virus.

## Abbreviations

IFN-α: Interferon-alpha; HCV: Hepatitis C virus; PBMCs: Peripheral blood mononuclear cells; ISGs: Interferon stimulated genes; SVR: Sustained virological response; G1: Genotype 1; G3: Genotype 3; RVR: Rapid virological response; EVR: Early virological response; EOT: End of treatment; DAAs: Direct acting antivirals; PI: Protease inhibitor; AUC: Area under the curve; PPV: Positive predictive value; NPV: Negative predictive value.

## Competing interests

The authors declare that they have no competing interests.

## Authors’ contributions

NMB, NJS and COF designed the experiments and wrote the manuscript. NB and SS performed experiments. NB, MTON, SS, NJS and COF discussed and interpreted the data. MTON, SS, SN and JH provided clinical insight, discussed and interpreted the data and reviewed the manuscript. COF directed the study. All authors read and approved the final manuscript.

## Supplementary Material

Additional file 1 **Figure S1.** Correlation between RVR and ISG expression. PBMCs were stimulated *in vitro* with 100 IU or 1000 IU of IFN-α for 2 or 4 h. qRT-PCR was used to quantify ISG expression. Analysis stratified into those who achieved RVR and those who did not. (A) all viral genotypes, (B) g1 infected patients and (C) g3 infected patients. All gene expression was normalised to expression of RPS15 and expressed on a log scale. Mann-Whitney statistical analysis, *p<0.05. **Figure S2.** Correlation between EVR and ISG expression. PBMCs were stimulated *in vitro* with 100 IU or 1000 IU of IFN-α for 2 or 4 h. qRT-PCR was used to quantify ISG expression. Analysis stratified into those who achieved EVR and those who did not. (A) all viral genotypes (B) g1 infected patients and (C) g3 infected patients. All gene expression was normalised to expression of RPS15 and expressed on a log scale. Mann-Whitney statistical analysis, *p<0.05. **Figure S3.** Correlation between EOT response and ISG expression. PBMCs were stimulated *in vitro* with 100 IU or 1000 IU of IFN-α for 2 or 4 h. qRT-PCR was used to quantify ISG expression. Analysis stratified into those who were PCR –ve at EOT and those who were PCR +ve. (A) all viral genotypes, (B) g1 infected patients and (C) g3 infected patients. All gene expression was normalised to expression of RPS15 and expressed on a log scale. Mann-Whitney statistical analysis, *p<0.05, **p<0.01. **Table S1.** Predictability of SVR based on cut-off gene expression values. Gene cut-off values quantified following 4h *in vitro* IFN-α stimulation, as determined by qRT-PCR, were analysed to predict response of g1 infected patients to respond to therapeutic IFN-α using receiver operating characteristic (ROC) analysis. AUC: area under curve in ROC analysis, CI: confidence intervals, PPV: positive predictive value, NPV: negative predictive value. Click here for file
