# Supplementary material for: In vitro blood cell responsiveness to IFN-α predicts clinical response independently of IL28B in hepatitis C virus genotype 1 infected patients
Source: J Transl Med. 2014 Jul 21;12:206. doi: 10.1186/1479-5876-12-206 (PMC4112837; doi:10.1186/1479-5876-12-206)
Supplement: Additional file 1 — Figure S1. Correlation between RVR and ISG expression. PBMCs were stimulated in vitro with 100 IU or 1000 IU of IFN-α for 2 or 4 h. qRT-PCR was used to quantify ISG expression. Analysis stratified into those who achieved RVR and those who did not. (A) all viral genotypes, (B) g1 infected patients and (C) g3 infected patients. All gene expression was normalised to expression of RPS15 and expressed on a log scale. Mann-Whitney statistical analysis, *p<0.05. Figure S2. Correlation between EVR and ISG expression. PBMCs were stimulated in vitro with 100 IU or 1000 IU of IFN-α for 2 or 4 h. qRT-PCR was used to quantify ISG expression. Analysis stratified into those who achieved EVR and those who did not. (A) all viral genotypes (B) g1 infected patients and (C) g3 infected patients. All gene expression was normalised to expression of RPS15 and expressed on a log scale. Mann-Whitney statistical analysis, *p<0.05. Figure S3. Correlation between EOT response and ISG expression. PBMCs were stimulated in vitro with 100 IU or 1000 IU of IFN-α for 2 or 4 h. qRT-PCR was used to quantify ISG expression. Analysis stratified into those who were PCR –ve at EOT and those who were PCR +ve. (A) all viral genotypes, (B) g1 infected patients and (C) g3 infected patients. All gene expression was normalised to expression of RPS15 and expressed on a log scale. Mann-Whitney statistical analysis, *p<0.05, **p<0.01. Table S1. Predictability of SVR based on cut-off gene expression values. Gene cut-off values quantified following 4h in vitro IFN-α stimulation, as determined by qRT-PCR, were analysed to predict response of g1 infected patients to respond to therapeutic IFN-α using receiver operating characteristic (ROC) analysis. AUC: area under curve in ROC analysis, CI: confidence intervals, PPV: positive predictive value, NPV: negative predictive value. [file 1479-5876-12-206-S1.pdf]

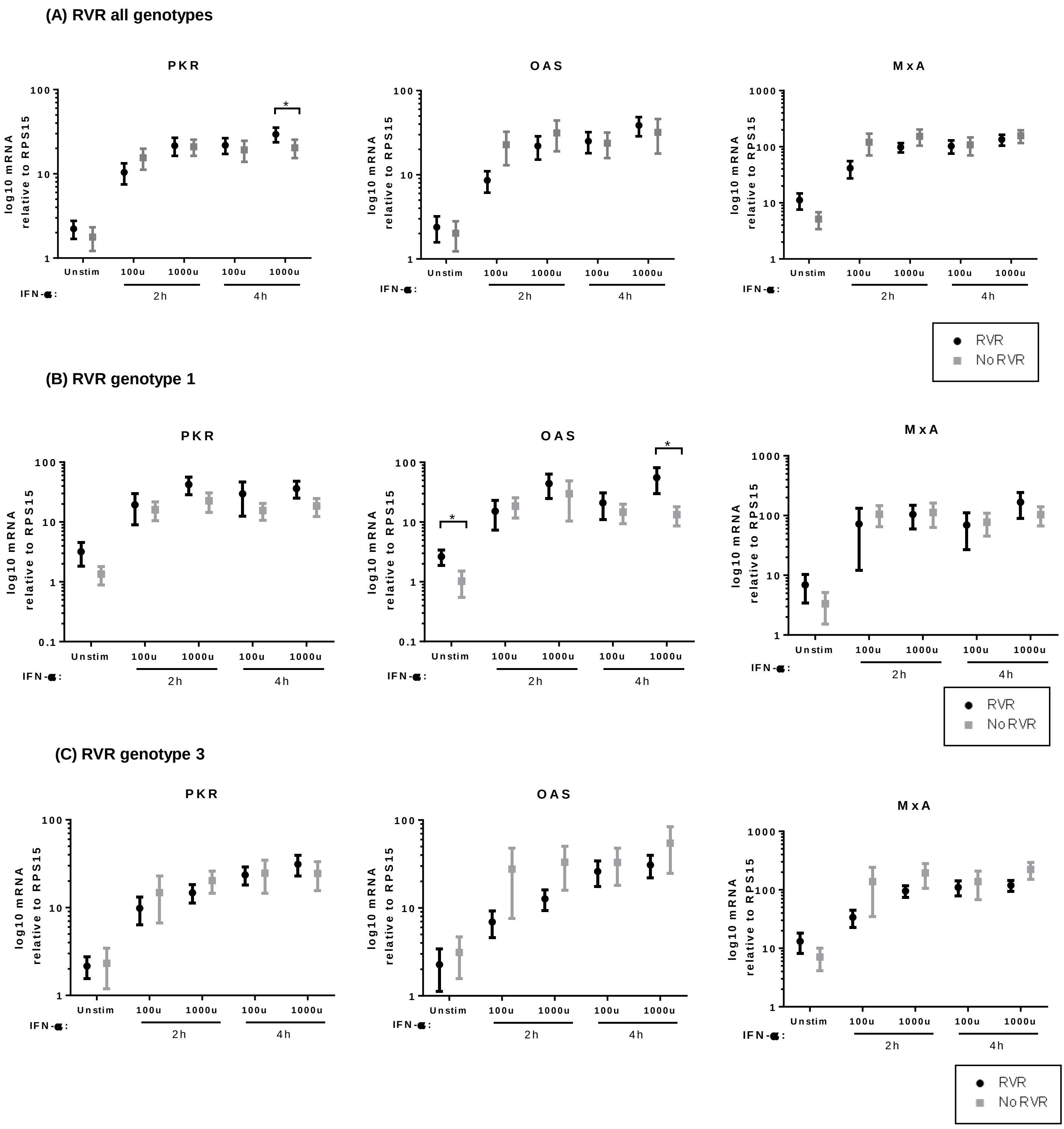

Supplemental Figure 1. Correlation between RVR and ISG expression. PBMCs were isolated from HCV infected patients prior to them commencing IFN-α therapy and were stimulated *in vitro* with 100IU or 1000IU of IFN-α for 2 or 4h. qRT-PCR was used to quantify ISG expression. Analysis stratified into those who achieved RVR and those who did not. (A) all viral genotypes, (B) genotype 1 infected patients and (C) genotype 3 infected patients. All gene expression was normalised to expression of the internal control gene RPS15 and expressed on a log scale. Mean and standard error of the mean (SEM) shown. Mann-Whitney statistical analysis, *$p<0.05$.

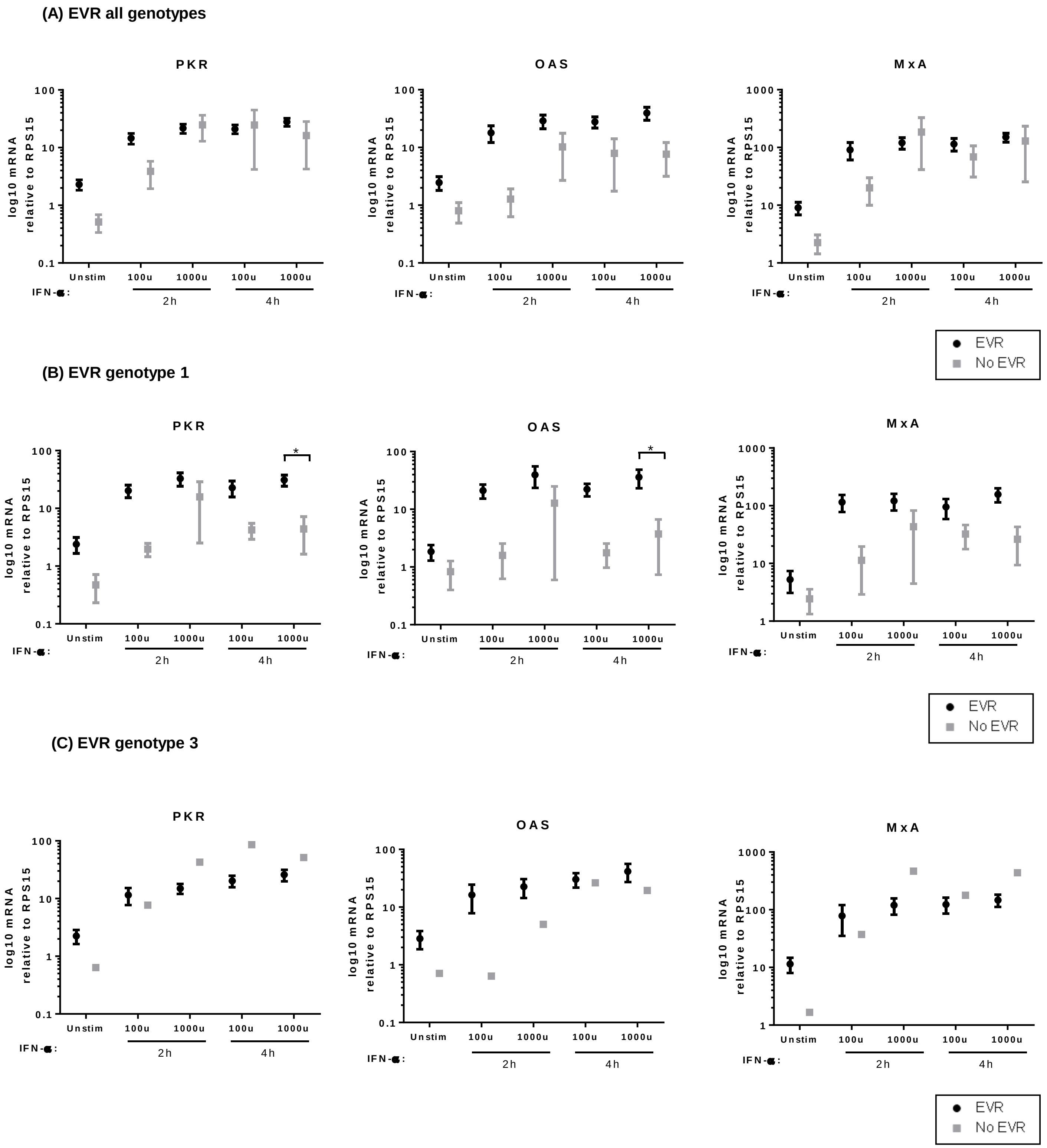

Supplemental Figure 2. Correlation between EVR and ISG expression. PBMCs were isolated from HCV infected patients prior to them commencing IFN-α therapy and were stimulated *in vitro* with 100IU or 1000IU of IFN-α for 2 or 4h. qRT-PCR was used to quantify ISG expression. Analysis stratified into those who achieved EVR and those who did not. (A) all viral genotypes (B) genotype 1 infected patients and (C) genotype 3 infected patients. All gene expression was normalised to expression of the internal control gene RPS15 and expressed on a log scale. Mean and standard error of the mean (SEM) shown. Mann-Whitney statistical analysis, *p<0.05.

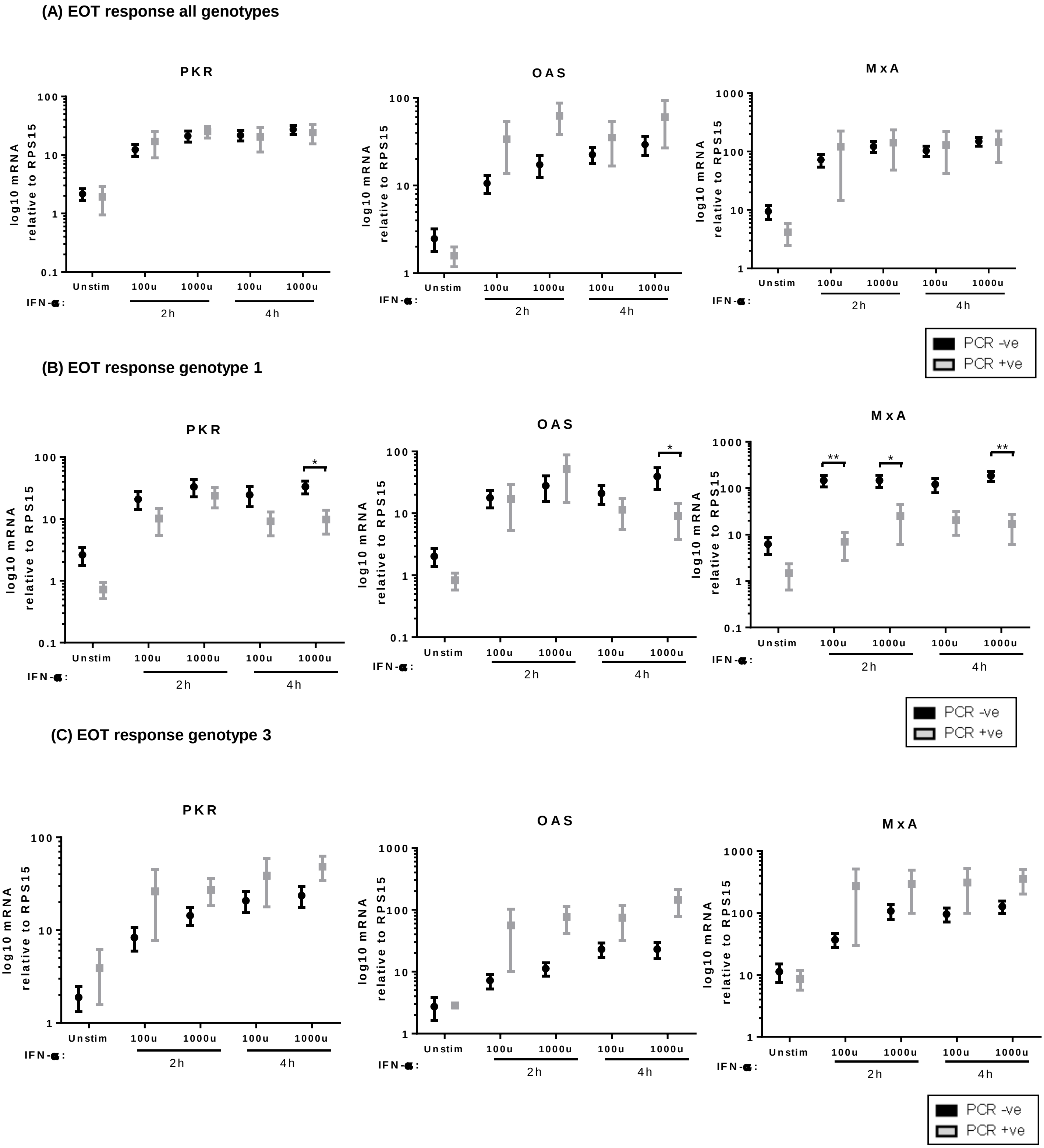

Supplemental Figure 3. Correlation between EOT response and ISG expression. PBMCs were isolated from HCV infected patients prior to them commencing IFN-α therapy and were stimulated *in vitro* with 100IU or 1000IU of IFN-α for 2 or 4h. qRT-PCR was used to quantify ISG expression. Analysis stratified into those who were PCR –ve at EOT and those who were PCR +ve. (A) all viral genotypes, (B) genotype 1 infected patients and (C) genotype 3 infected patients. All gene expression was normalised to expression of the internal control gene RPS15 and expressed on a log scale. Mean and standard error of the mean (SEM) shown. Mann-Whitney statistical analysis, $*p<0.05$, $**p<0.01$.

Variables	AUC	95% CI	Cut-Off	Sensitivity (%)	Specificity (%)	PPV (%)	NPV (%)	Likelihood ratio
PKR	0.86	0.67-1.1	27.56	100	62.5	100	72.7	2.7
OAS	0.94	0.82-1.1	31.92	100	62.5	100	72.7	2.7
MxA	0.94	0.82-1.1	91.72	100	87.5	100	88.9	8

Supplemental Table 1. Predictability of SVR based on cut-off gene expression values

Gene cut off values quantified following 4h *in vitro* IFN-α stimulation, as determined by qRT-PCR, were analysed to predict response of g1 infected patients to respond to therapeutic IFN-α using receiver operating characteristic (ROC) analysis. AUC: area under curve in ROC analysis, CI: confidence intervals, PPV: positive predictive value, NPV: negative predictive value.
